# A novel frameshift deletion in autosomal recessive *SBF1*-related syndromic neuropathy with necklace fibres

**DOI:** 10.1007/s00415-020-09827-y

**Published:** 2020-05-22

**Authors:** Qiang Gang, Conceição Bettencourt, Janice Holton, Christopher Lovejoy, Viorica Chelban, Emer Oconnor, Yun Yuan, Mary M. Reilly, Michael Hanna, Henry Houlden

**Affiliations:** 1 Department of Neurology, Peking University First Hospital, Beijing, 100034 People’s Republic of China; 2 Department of Neuromuscular Diseases, UCL Queen Square Institute of Neurology, Queen Square, London, WC1N 3BG UK; 3 MRC Centre for Neuromuscular Diseases, UCL Queen Square Institute of Neurology, Queen Square, London, WC1N 3BG UK; 4 The Queen Square Brain Bank for Neurological Disorders, UCL Queen Square Institute of Neurology, Queen Square, London, WC1N 3BG UK; 5 Department of Clinical and Movement Neurosciences, UCL Queen Square Institute of Neurology, Queen Square, London, WC1N 3BG UK; 6 Department of Neurodegenerative Disease, UCL Queen Square Institute of Neurology, Queen Square, London, WC1N 3BG UK; 7 Neurogenetics Laboratory, UCL Queen Square Institute of Neurology, Queen Square, London, WC1N 3BG UK

**Keywords:** *SBF1*, MTMR5, CMT4B, Necklace fibres

## Abstract

**Objective:**

To identify the genetic cause of complex neuropathy in two siblings from a consanguineous family.

**Methods:**

The patients were recruited from our clinic. Muscle biopsy and whole-exome sequencing (WES) were performed. Fibroblasts cell lines from the index patient, unaffected parents, and three normal controls were used for cDNA analysis and western blot.

**Results:**

The index patient was a 29-year-old male with clinical phenotype of syndactyly, pes cavus, swallowing difficulties, vision problem, imbalance, and muscle weakness. The sibling had similar, but milder symptoms. Nerve conduction studies and electromyography of both patients suggested sensory-motor axonal neuropathy. Muscle biopsy showed a feature of necklace fibres. WES identified a novel homozygous frameshift deletion (c.5477-5478del; p.1826-1826del) in exon 40 of the *SBF1* gene in the two siblings, while both parents and the unaffected sibling were heterozygous carriers. Functional analysis showed a markedly reduced level of MTMR5 protein encoded by *SBF1* in the index case. The levels of MTMR5 protein in unaffected parents were similar to those found in controls.

**Conclusion:**

A novel homozygous frameshift deletion in *SBF1* was identified in this family. Sensory-motor axonal neuropathy and necklace fibres in biopsy were the major features expanding the phenotypic spectrum of *SBF1*-related recessive syndromic neuropathy.

**Electronic supplementary material:**

The online version of this article (10.1007/s00415-020-09827-y) contains supplementary material, which is available to authorized users.

## Introduction

Charcot–Marie–Tooth (CMT) disease is a large group of clinically and genetically heterogeneous inherited neuropathies which are characterised by slowly progressive distal muscle weakness, wasting, and sensory loss. Autosomal recessive forms of demyelinating CMT are also called CMT4 and a specific subgroup CMT4B is characterised by severe demyelinating neuropathy with focal myelin outfoldings and secondary axonal loss [1]. Mutations in genes encoding proteins of myotubularin family, namely *MTMR2* (OMIM#603557) [2], *MTMR13*/*SBF2* (OMIM#607697) [3], and *MTMR5*/*SBF1* (OMIM#603560) [4], are associated with subtypes CMT4B1, CMT4B2, and CMT4B3, respectively. In addition to the pure neuropathic form of CMT4B3, mutations in *SBF1* have also been reported in four families with severe syndromic manifestations such as sensory-motor polyneuropathy, multiple cranial nerve involvement, cognitive delay, and skeletal abnormalities [5–7]. In this study, a novel frameshift deletion in *SBF1* was identified in two siblings with complex neuropathy and “necklace fibres” in muscle biopsy, expanding the spectrum of *SBF1*-related syndromic neuropathy.

## Methods

**Fig. 1:**
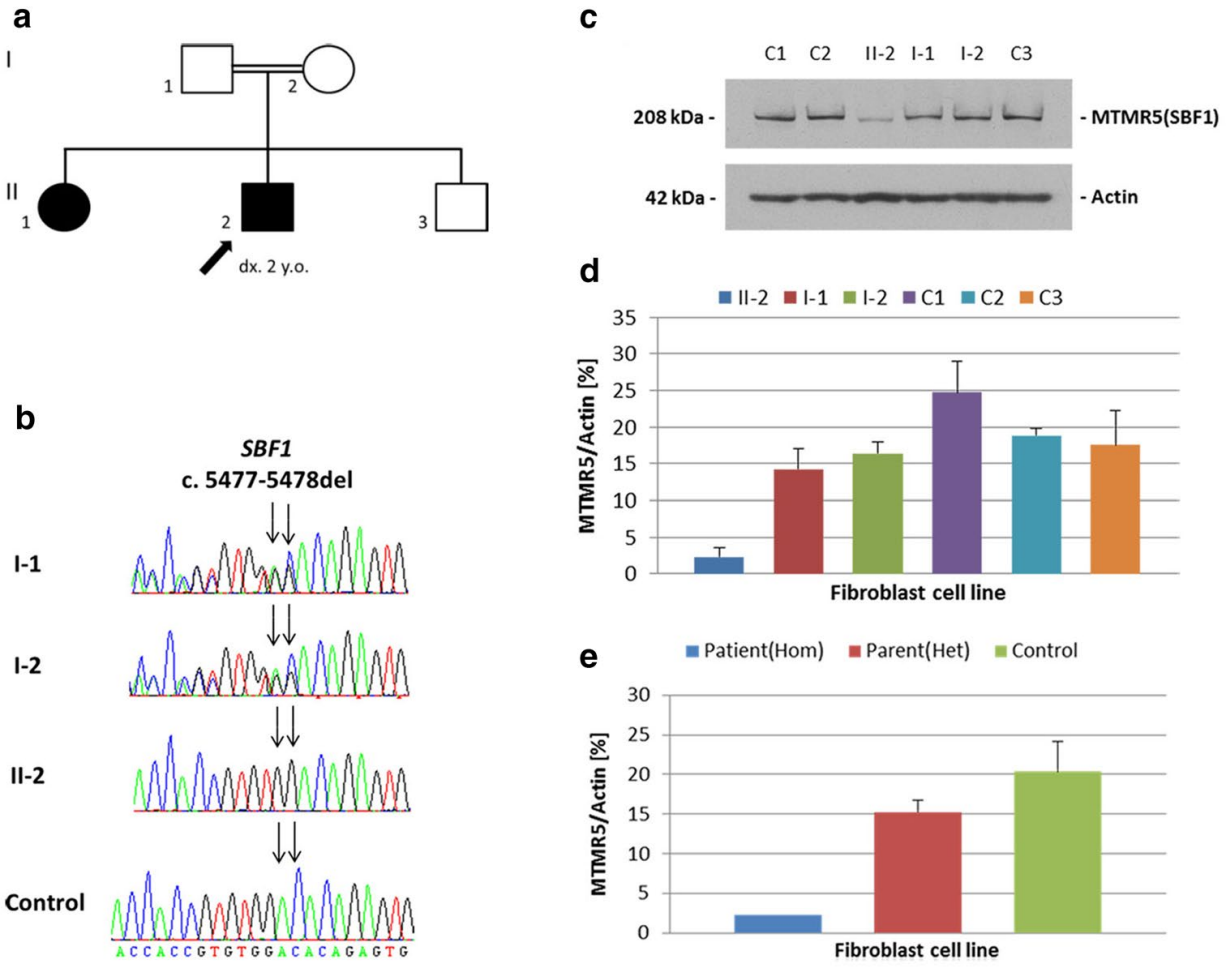
Pedigree of the family with two affected siblings and genetic phenotypes in the family. **a** Pedigree of the family (black symbols represent affected patients; black arrow points the index patient of the family); **b** Electropherograms of cDNA for the frameshift deletion in the *SBF1* gene (I-1 and I-2 are parents; II-2 is the index patient; control is a normal healthy control); **c** MTMR5 and actin antibody in fibroblasts (this represents an example of three experiments carried out); **d**–**e** Expression level of MTMR5 protein in fibroblasts from the family compared with normal controls (error bar represents the standard deviation; C1–C3 are three normal controls. *Hom* homozygous, *Het* heterozygous.)

The affected siblings from a British family (II-2 and II-1, Fig. 1a) were seen and examined by neurologists at the National Hospital for Neurology and Neurosurgery (NHNN), University College London (UCL). Muscle biopsy was performed on the index case (II-2). MRI scans were performed in both patients. Whole-exome sequencing was performed on the DNA from the index case (II-2). The data were processed through an in-house bioinformatics pipeline as described previously [8]. Fibroblasts cell lines obtained from skin biopsies from the index case, unaffected parents (I-1 and I-2), and three normal controls (C1–C3) were used for mRNA analysis and western blot.

## Results

**Fig. 2:**
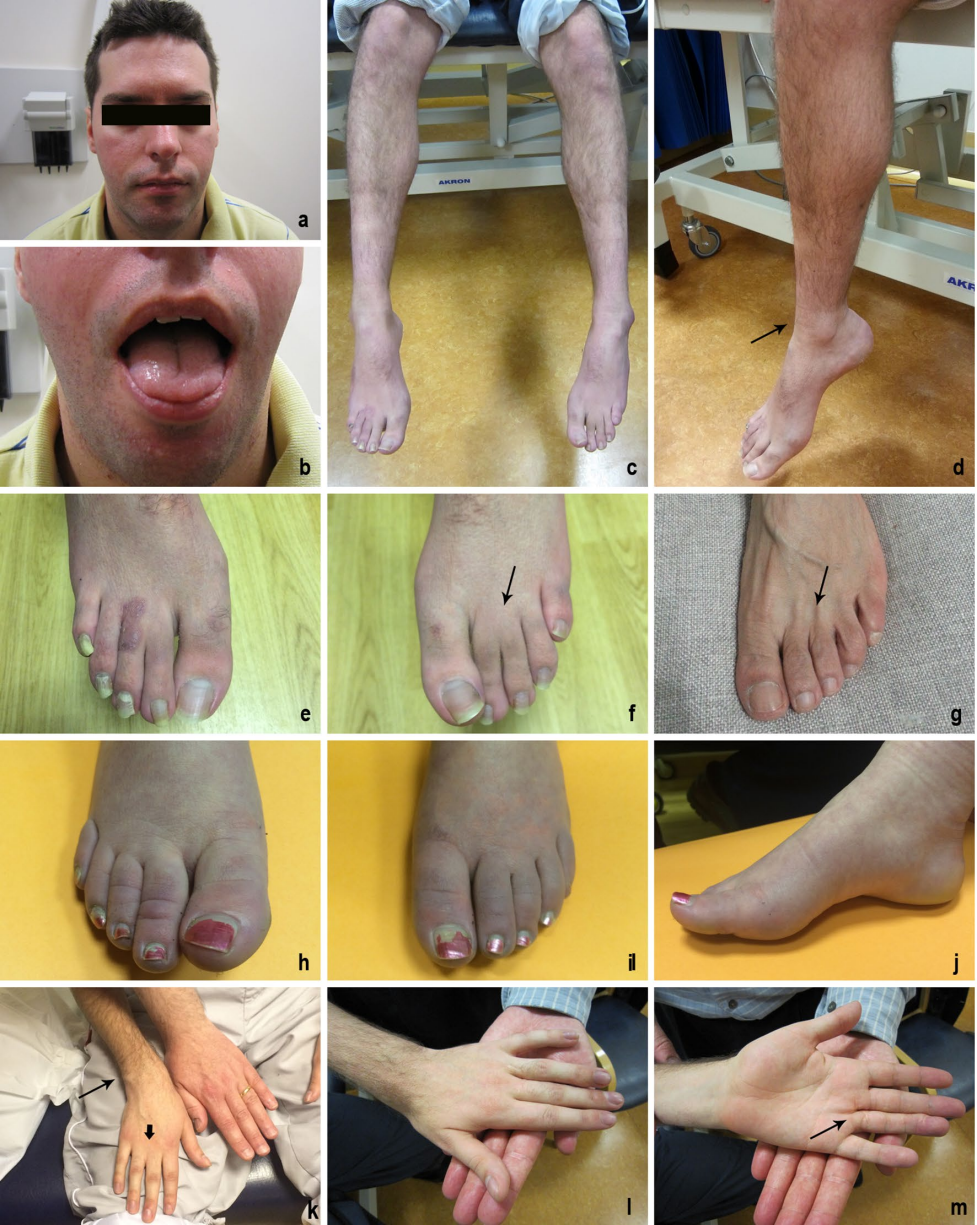
Clinical features of the index patient and his sister. **a**–**f** and **k**–**m** were from the index patient II-2, and **h**–**j** were from the affected sister II-1. The tongue of the index patient was atrophic (**b**); muscle wasting in both calves (**c**); pes cavus and slim ankle as pointed by arrow in (**d**); syndactyly of two toes of both feet (**e**–**f**) as pointed by arrow, compared to a normal foot (**g**); abnormal colour of both feet of the affected sister (**h**–**j**); syndactyly of two fingers of both hands (**k**–**m**) pointed by a small arrow in compared to a normal hand on the right (**k**), a slim wrist pointed by an arrow in (**k**), and a surgery scar on left hand pointed by arrow in (**m**)

**Fig. 3:**
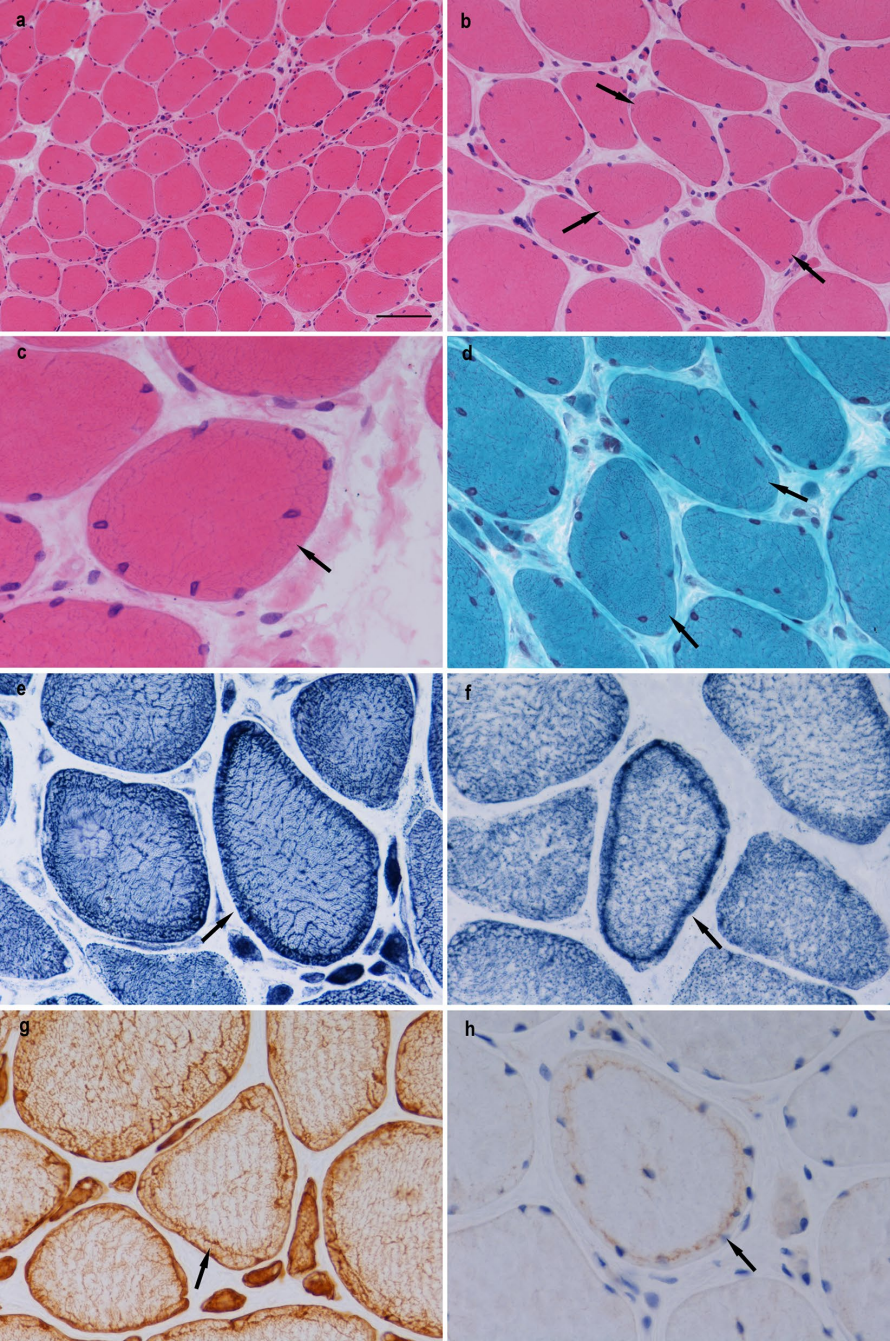
Biopsy features of the index patient. H&E staining showed an increased variation in fibre size and frequent internal nuclei (**a**); internal nuclei were often distributed in a linear fashion displaced within the fibre and laying along a basophilic line resembling a necklace fibre (arrows in **b** and **c**); Gomori trichrome staining showed some increase in the density of mitochondrial staining in a peripheral band in occasional fibres (arrow in **d**), and there were no ragged red fibres; NADH-TR (**e**) and SDH (**f**) staining confirmed the impression of necklace fibres with an increase in the intensity of peripheral staining with a band-like pattern; (**g**) showed some desmin-positive staining in the peripheral band of many fibres; and (**h**) showed fine granular p62-positive staining along the ring visible in a necklace fibre. Necklace fibres are indicated by arrows. Scale bar represents 100 μm in **a**; 50 μm in **b** and **d**; 25 μm in **c** and **e**–**h**

**Fig. 4:**
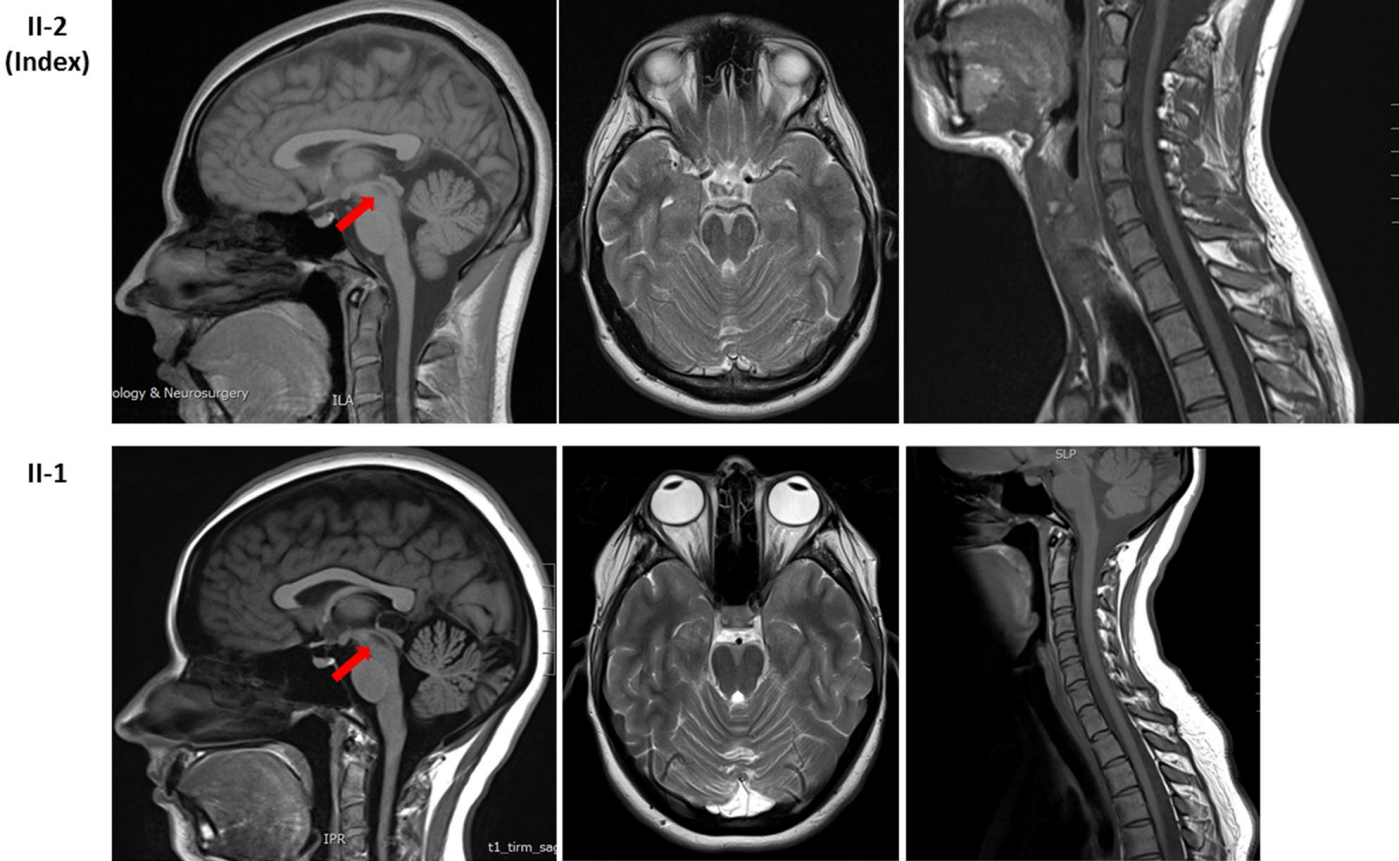
Brain and spinal cord MRI of the siblings. Upper lane: a focus mature damage in the periaqueductal grey matter of the upper tegmentum was observed in the index patient (II-2) (pointed by the red arrow). The cord was mildly thinned throughout. Lower lane: the sibling (II-1) showed mild volume loss of the lateral folia of both cerebellar hemispheres in keeping with mild cerebellar atrophy; there is abnormality in the posterior and middle area of the midbrain (pointed by the red arrow), involving symmetrically the medial longitudinal fasciculus and extending into the red nuclei; the diameter of spinal cord is smaller than expected which indicates some grade of cord atrophy

The index patient (II-2) and his affected sister (II-1) were from a consanguineous family where parents are second cousins (Fig. 1a). The patient II-2 was born with syndactyly of the third and fourth fingers of both hands and the second and third toes of both feet. He also had pes cavus of both feet (Fig. 2d–f). He started walking by age of 1^1^/_2_, and his walking became clumsy at the age of three. By the age of four, he also had difficulty in pronouncing consonants. He was noted to display poor pencil grasp at the age of eight, and later received a diagnosis of dyspraxia with hypotonia. In addition, he had visual abnormalities with bilateral dilated pupils (unreactive to light) at the age of two, and had a history of photophobia dating back to the age of three. Over the years, the patient developed symptoms of slowness of swallowing, choking on food, blurred and double vision, imbalance, dragging his legs, easy fatigue, and weakness. These symptoms, particularly his gait, speech, and eyelid drop, were worse in the evening. The patient currently wears ankle foot to support his walking. On examination, he had obvious strabismus, gaze-evoked nystagmus to the left, as well as on up-gaze, and he had broken pursuit movements. He has a tonic pupil not reactive to light or accommodation. His fundi were normal. The rest of cranial nerve examination was normal. Power in lower limbs was Grade 4/5 throughout. Distal weakness in wrist extension was at 4/5, and finger extension and flexion were at 3/5. He displayed symmetrical hypo-reflexia with bilateral extensor plantars; however, his coordination was normal. Sensory examination showed that he had a patchy pin-prick loss in a glove and stocking distribution. Blood tests were negative for acetylcholine receptor antibodies, anti-MUSK antibody, and anti-voltage gated calcium channel antibody. The creatine kinase level and metabolic testing were normal. Electromyography and nerve conduction study of both patients were performed, and showed moderately severe axonal sensory-motor neuropathy (Table 1). There was no evidence of defect at the neuromuscular junction. There were mild chronic neurogenic changes on sampling the left orbicularis oculi. MRI of his head and whole spine showed mature damage in periaqueductal grey matter of upper tegmentum, and the prominence of the central canal at T7/8 level and the cord was mildly thinned throughout, but was otherwise normal (Fig. 4). Muscle biopsy showed marked neurogenic atrophy with prominent internal nuclei and appearance of necklace fibres (Fig. 3).

**Table 1:** Electrophysiological assessment of the patients

Case	Motor nerve conduction studies						Sensory nerve conduction studies						EMG		Summary findings of NCS and EMG
	Median nerve			Tibial nerve			Median nerve		Ulnar nerve		Sural nerve		Limbs Upper:lower		
	DML (msec)	CMAP amp (mV)	MCV (m/s)	Distal lat (msec)	CMAP amp (mV)	MCV (m/s)	SNAP amp (uV)	SCV (m/s)	SNAP amp (uV)	SCV (m/s)	SNAP amp (uV)	SCV (m/s)	Chronic denervation		
II-2 (index)	3.8	5.6	54	4	**1.7**	40	10	57	6	51	**3**	36	Yes	Yes	Sensory-motor axonal neuropathy
II-1	4	3.6	55	6.1	**1.6**	48	2	53	2	51	**NR**	51	Yes	Yes	Sensory-motor axonal neuropathy

*NR* no response, *Amp* amplitude, *lat* latency, *DML* distal motor latency, *CMAP* Compound Muscle Action Potential , *V* microvolts, *m/s* metres per second, *msec* milliseconds

The elder sister (II-1) of the index patient also had similar pupils and disc appearance to the index patient, with slightly difficult walking, some weakness and wasting in her hands and feet, and endometriosis. On examination, her eyes showed slightly jerky pursuit movement. She had thin wrists and small hands but no clear wasting and only slight weakness of the thumbs. Her feet were similar to the hands, with only minimal weak dorsiflexion. She had similar sensory loss in a glove and stock distribution, and her feet also became discoloured, often black and quite cold. (Fig. 2h–j) She also showed similar changes in MRI of brain and spinal cord (Fig. 4).

Both of their parents and the younger brother were not affected. Overall, the phenotype in this family is consistent with an autosomal recessive form of inheritance.

During genetic analysis, we screened for nonsynonymous, splice-site and coding indel variants with a minor allele frequency (MAF) < 1% in the 1000 Genomes project, Exome Variant Server, and the Exome Aggregation Consortium (ExAC) databases. In the filtering steps, we also prioritised for homozygous or compound heterozygous variants in autosomal recessive genes. This left a novel homozygous frameshift deletion (c.5477-5478del; p.1826-1826del) in exon 40 of the *SBF1*/*MTMR5* gene, a known gene for CMT. This deletion is absent in the above public databases. The variant were confirmed by Sanger sequencing. Segregation analysis in both parents and two siblings found that the affected sister (II-1) also harbours the same homozygous frameshift deletion, and both parents as well as the unaffected sibling are heterozygous carriers of the deletion in *SBF1* (Fig. 1b). The deletion occurs in the last exon of *SBF1* gene and causes a frameshift. This leads to a premature stop codon at 1850 amino acid position and produces a truncated protein by 43 amino acid residues. Western blot showed a markedly reduced level of MTMR5 protein in the patient with homozygous frameshift deletion compared with both heterozygous parents and controls. (Fig. 1c–e). There was a trend that the level of MTMR5 protein was lower in carriers than controls. This suggests that the homozygous frameshift deletion p.1826-1826del in *SBF1* causes a marked decay of the protein level.

## Discussion

Our family is the sixth family identified with mutations in *SBF1* (Supplementary Table). They share several similar phenotypes with previously reported families with CMT4B3. Ophthalmoplegia was seen in most reported cases in addition to progressive weakness of the limbs and sensory-motor axonal neuropathy [5, 9]. Development abnormalities were also common features, such as short stature, microcephaly, syndactyly, scoliosis, lumbar hyperlordosis, foot drop, pes cavus/planus, and gynecomastia. However, microcephaly and moderate-to-severe intellectual disability were observed in three previously described families [5, 7, 10], but not in the present family. The distinct “fork and bracket” sign at the pontine and mesencephalic level of brain MRI was reported in two previous families [7, 10], while the MRI of our patients showed abnormality in the posterior area of the midbrain and spinal cord atrophy. Although the EMG suggested a feature of axonal sensory-motor neuropathy, the appearance of necklace fibres seen in muscle biopsy is an intriguing feature observed in our index patient, which was not observed in the previous *SBF1*-related cases. Necklace fibres with nuclear internalisation have been reported as a histological hallmark of X-linked myotubular myopathy related to mutations in the phosphoinositide phosphatase myotubularin 1 (*MTM1*) gene and also a late-onset dynamin-2 (*DNM2*) gene-related centronuclear myopathy [11, 12]. These fibres were usually described to be small and obliquely oriented with an increased density of mitochondria and sarcoplasmic reticulum profiles, and internal nuclei usually aligned like the necklace [13]. Neither *MTM1* nor *DNM2* was found in the index patient. Our study is the first to report necklace fibres in *SBF1*-related phenotypes, which expands the clinical and molecular genetic spectrum of this specific pathological feature.

The *SBF1* gene encodes protein MTMR5, a member of the myotubularin-related (MTMR) family. MTMR proteins are a large family of phosphoinositide (PI) 3-phosphatases which are involved in membrane trafficking and endocytosis [14]. The protein domain cores include GRAM domain (Glucosyltransferase, Rab-like GTPase Activator, and Myotubularins), RID (Rac-Induced recruitment Domain), PTP/DSP homology (tyrosine/dual-specificity phosphatase super-family), and SID (SET-interacting domain) [14]. Some members of the family contain a catalytically active PTP/DSP domain, such as MTMR2 [14]. Some members contain catalytically inactive substitutions in the phosphatase domain, and thus, they belong to a subgroup called “pseudophosphatases”, such as MTMR5 and MTM13 [14]. Studies suggested that MTMR5 and MTMR13 interact with MTMR2 directly via their coiled-coil domains increasing the phosphatase activity of MTMR2 as well as commanding its subcellular localization [14].

Loss of function of MTMR5 was suggested to cause a defect in the late stages of spermatogenesis in mice [15] and studies on humans showed that rare variants in *SBF1* are linked to male infertility [16]. However, the pathophysiological pathways of MTMR5-induced neuropathy have not been fully elucidated. MTMR5 and MTMR13 are homologues which share 59% overall sequence identity. It has been suggested that loss of function of MTMR13, encoded by the *SBF2* gene, leads to alterations in MTMR2 function and subsequent dysfunction of membrane trafficking, endocytic/exocytic processes, and neuron–Schwann cell interactions [17]. MTMR13 -/- mice showed myelin outfoldings and infoldings in motor and sensory peripheral nerves as pathological hallmarks of CMT4B2 [18]. The level of MTMR2 was decreased by ~ 50% in MTMR13-/- sciatic nerves, suggesting that MTMR13 influences MTMR2 stability [18]. MTMR2 also plays a crucial role in axon development and maintenance by interacting with the neurofilament light chain [19]. This could be affected due to altered function of MTMR13.

As MTMR5 is the closest homologue of MTMR13, it is possible that the loss of function in MTMR5 could also induce dysfunction in neuronal or Schwann cells via a similar mechanism with the MTMR2/MTMR13 complex. This may explain the strikingly similar phenotypes of patients with recessive mutations in the *MTMR2*, *SBF2,* and *SBF1* genes.

In the present study, the protein level of MTMR5 was markedly reduced due to the homozygous frameshift deletion in *SBF1* identified in both affected siblings, indicating a loss-of-function mechanism underlying the pathogenesis of the disease. Whether this causes the patient to be infertile is as yet unknown.

## Conclusion

We reported a novel homozygous frameshift deletion in *SBF1* in a family affected with a rare form of neuropathy with necklace fibres on the muscle biopsy. Segregation and functional analysis suggested that the homozygous deletion in *SBF1* was the main culprit to the disease via a loss-of-function mechanism. Our findings in this family expand the phenotypic spectrum of *SBF1*-related recessive syndromic neuropathy.

## Electronic supplementary material

Below is the link to the electronic supplementary material.

Supplementary file1 (DOCX 25 kb)

## Data Availability

Yes.
