# Supplementary material for: A novel frameshift deletion in autosomal recessive SBF1-related syndromic neuropathy with necklace fibres
Source: J Neurol. 2020 May 22;267(9):2705–12. doi: 10.1007/s00415-020-09827-y (PMC7419361; doi:10.1007/s00415-020-09827-y)
Supplement: Supplementary file 1 — Supplementary file1 (DOCX 25 kb) [file 415_2020_9827_MOESM1_ESM.docx]

**Supplementary Table. Detailed phenotypic comparison of the four *SBF1*-related families**

|  | **Present study** | **Present study** | **Nakhro et al., 2013** | **Alazami et al., 2014** | **Romani et al., 2016** | **Manole et al., 2016** | **Flusser et al., 2018** |
| --- | --- | --- | --- | --- | --- | --- | --- |
| **Patients (sex)** | II-2 (M) | II-1 (F) | 3 sibs (3F) | 3 sibs (3M) | 2 sibs (1M/1F) | 2 sibs (2M) | 4 sibs (3M/1F) |
| **Ethnicity** | British | British | Korean | Saudi Arabian | Syrian | Spanish | Israeli Bedouins |
| **Genetics**  **cDNA change**  **Protein change**  **MAF ExAC**  **PolyPhen** | ***SBF1***  c.5477-5478del (hom)  p.1826-1826del (hom)  -  N/A | ***SBF1***  c.5477-5478del (hom)  p.1826-1826del (hom)  -  N/A | ***SBF1***  c.1249A>G (het)  c.4768A>G (het)  p.M417V (het)  p.T1590A (het)  0.00005/0.00032  Tolerated/Tolerated | ***SBF1***  c.1327G>A (hom)  p.D443N (hom)  -  Damaging | ***SBF1***  c.1004T>C (hom)  p.L335P (hom)  -  Damaging | ***SBF1***  c.1168C>G (het)  c.2209_2210del (het)  p.R390G (het)  p.L737Efs*3 (het)  -/-  Probably damaging/- | ***SBF1***  c.1636+1G>A (hom)  -  N/A |
| **Age of onset**  **Age at examination** | Infancy  29 | Childhood  30 | 11/5/7  57/54/52 | Infancy  46/45/34 | Infancy  23/18 | 4/9  48/49 | Infancy  Infancy -  mid-20s |
| **First symptoms** | Clumsy walking | unknown | Distal leg weakness, gait ataxia | Strabismus, cognitive delay | Microcephaly | Gait difficulties | Microcephaly, pyramidal signs |
| **Cranial nerves**  **Ophthalmoplegia**  **Strabismus**  **Nystagmus**  **Tonic pupil**  **Visual abnormalities**  **Hearing loss**  **Facial weakness**  **Dysphagia**  **Dysarthria**  **Gag reflex**  **Neck flexion**  **Tongue involvement** | Yes  Yes  Yes  Yes  No  No  No  Yes  No  -  No  Atrophy | Yes  No  No  No  No  No  No  No  No  -  No  No | -  -  -  -  -  -  -  -  -  -  -  - | Yes  Yes  -  Yes (1)  -  -  Yes  Yes (1)  Mild (1)  -  -  - | Yes  Yes  -  Yes  Yes  -  Yes  Yes  Yes  -  -  - | Yes  No  Horizontal gaze-evoked nystagmus  -  -  Yes, bilateral  Yes, bilateral, asymmetric  Yes  Yes  Absent/ diminished  Mild weakness  Weakness, atrophy | Yes  Yes  Yes  -  -  -  Yes  Yes  Yes  -  -  No |
| **Muscle weakness**  **Upper limb**  **Lower limb**  **Muscle atrophy** | Yes, distal > proximal  Yes, moderate – severe  Yes, moderate | Yes, mild distal  Yes, mild  Yes, mild | Yes  Yes  Yes | Yes  Yes  Yes | Yes, distal > proximal  Yes  Yes | Yes, distal > proximal  Yes, severe  Yes, moderate | Yes, distal > proximal |
| **Sensory loss** | Yes | Yes | Yes | Yes (2) | - | Yes | No |
| **Deep tendon reflexes** | Absent | Absent | Absent | Absent | Absent | Absent | Absent |
| **Development abnormalities**  **Short stature**  **Microcephaly**  **Skeletal anomalies**  **Foot drop**  **Pes cavus/ planus**  **Others** | a  Yes  No  Syndactyly  Yes  Pes cavus | A  Yes  No  No  No  Pes cavus | A  -  -  Scoliosis (2)  -  Pes planus | A  Yes (2)  Yes  Syndactyly  Yes  - | A  -  Yes  Mild joint laxity, thumb sign  Yes  Pes cavus  Facial dysmorphic features | A  No  No  Lumbar hyperlordosis  No  Pes cavus  Gynecomastia | Yes (2)  Yes  Kyphoscoliosis  Yes  Pes cavus  Facial dysmorphic features, retrognathia, high arched palate and simple ears |
| **Unsteady gait** | High stepping gait and ataxic gait (difficult tandem walking) | Mild high stepping gait | Yes | - | Yes | Yes | Yes, ataxia |
| **Intellectual impairment** | No | No | - | Yes | Yes | Yes | - |
| **EMG & NCS** | Sensory-motor axonal neuropathy, severe | Sensory-motor axonall neuropathy, moderate | Sensory-motor neuropathy | Sensory-motor axonal polyneuropathy | Sensory-motor axonal neuropathy | Sensory-motor axonal neuropathy | Sensory-motor axonal neuropathy |
| **MRI** | Midbrain tegmentum and peiaqueductal grey lesion; mildly thinned cord | Mild cerebellar atrophy; abnormality in the posterior and middle area of the midbrain | Not performed | Brain atrophy | Fork and bracket sign at the pontine and mesencephalic level | Mild cerebellar atrophy | Cerebellar atrophy, fork and bracket sign, prominent ventricles and enlarged subarachnoid space |
| **Muscle/ nerve biopsy** | Muscle biopsy: neurogenic atrophy and necklace fibres.  Nerve not examined | Not examined | Muscle not examined. Nerve biopsy: focally folded myelin sheaths. | Muscle biopsy: neurogenic angulated fibres and mild fibre type grouping.  Nerve biopsy: moderately depleted myelinated axons of all fibre size with no onion bulbs. | Not examined | Muscle biopsy: longstanding neurogenic atrophy.  Nerve biopsy: diffuse loss of myelinated axons of all diameters, few regenerating clusters and occasional moderately thin myelin sheaths, with no onion bulbs. | Not examined |

Footnotes: F = female, M = male, sibs = siblings, hom = homozygous, het = heterozygous, N/A = not available, MAF = minor allele frequency, ExAC = Exome Aggregation Consortium, EMG = electromyography, NCS = nerve conduction study, MRI = magnetic resonance imaging
